# Cell Culture on Low-Fluorescence and High-Resolution Photoresist

**DOI:** 10.3390/mi11060571

**Published:** 2020-06-04

**Authors:** Hidetaka Ueno, Katsuya Maruo, Masatoshi Inoue, Hidetoshi Kotera, Takaaki Suzuki

**Affiliations:** 1 Division of Mechanical Science and Technology, Gunma University, Kiryu 376-8515, Japan; h-ueno@gunma-u.ac.jp; 2 Health and Medical Research Institute, National Institute of Advanced Industrial Science and Technology, 2217-14 Hayashi-cho, Takamatsu 761-0395, Japan; 3 Innovation Park, Daicel Corporation, Himeji 671-1283, Japan; kt_maruo@jp.daicel.com; 4 Division of Intelligent Mechanical Systems Engineering, Kagawa University, Takamatsu 761-0396, Japan; Masatoshi.Inoue@mitsuichemicals.com; 5 RIKEN, Wako 351-0198, Japan; hidetoshi.kotera@riken.jp

**Keywords:** low-fluorescence, high resolution, photoresist, cell analysis, cell chip, micro and nanotechnology

## Abstract

2D and 3D topographic cues made of photoresist, a polymer, are used for cell culture and cell analysis. Photoresists used for cell analysis provide the surface conditions necessary for proper cell growth, along with patterning properties of a wide range and high precision, and low auto-fluorescence that does not affect fluorescence imaging. In this study, we developed a thick negative photoresist SJI-001 possessing the aforementioned properties. We evaluated the surface conditions of SJI-001 affecting cell culture. First, we studied the wettability of SJI-001, which was changed by plasma treatment, conducted as a pretreatment on a plastic substrate before cell seeding. SJI-001 was more chemically stable than SU-8 used for fabricating the micro-electromechanical systems (MEMS). Furthermore, the doubling time and adhesion rate of adherent HeLa cells cultured on untreated SJI-001 were 25.2 h and 74%, respectively, thus indicating its suitability for cell culture over SU-8. In addition, we fabricated a cell culture plate with a 3D lattice structure, three micrometers in size, using SJI-001. HeLa cells seeded on this plate remained attached over five days. Therefore, SJI-001 exhibits surface conditions suitable for cell culture and has several bioapplications including microstructures and cell chips for cell culture and cell analysis.

## 1. Introduction

Photoresists are a type of polymer used for fabricating cell chips, such as BioMEMS and lab-on-a-chip, for cell culture and cell analysis due to its capability of fabricating a nanometer (nm) structure by simple fabrication processes [1,2,3,4,5,6,7,8]. Photoresists used for cell chips are required the patterning properties, surface conditions and low auto-fluorescence. Patterning properties is evaluated by the resolution and aspect ratio, wherein high resolution and high aspect ratio are preferred for fabricating finer and more complex structures [9]. Surface condition affects cells which are cultured on the photoresist, so it must be ensured that the photoresist should not affect cell phenotype both physically and chemically [10]. Low auto-fluorescence is also required for photoresists because the auto-fluorescence reduces the S/N ratio of fluorescence imaging [11]. To develop the photoresist which is suitable for these requests will directly contribute to BioMEMS and lab-on-a-chip research field.

SU-8, a negative type of photoresist, is commonly used for fabricating cell chips. Since SU-8 is capable of fabricating microstructures with an aspect ratio over five, it is used for fabricating microchannels or molds for soft lithography [12,13,14,15,16,17,18]. The size and shape of the structure is capable of being changed by designing mask pattern. In addition, some adherent cell cultures have been conducted on flat surfaces or microstructures made of SU-8 [19,20]. Therefore, SU-8 has exhibited patterning properties and surface conditions that are suitable for cell chips. However, since SU-8 has a benzene ring, which results in high auto-fluorescence, it proves disadvantageous for fluorescence imaging [21].

Our research group has developed SJI-001, a low-fluorescence photoresist, by changing the benzene ring to cyclohexane and antimony to phosphorus, as compared to the original SU-8 compound. It has been indicated that SJI-001 has lower auto-fluorescence compared to SU-8 and high patterning properties equivalent to SU-8 [22]. For example, the autofluorescence of the SJI-001 is 90% lower than SU-8 at 461-nm-emission light. SJI-001 can fabricate micropattern the aspect ratio of which is over 7. However, the surface conditions of SJI-001 and its effect on adherent cells had not been previously evaluated. Since adherent cells sense the environment and thus determine their adhesion, proliferation and necrosis, the effect of the material surface conditions on cells must be considered [23].

In this study, we evaluated the effect of the SJI-001 surface and its suitability as a scaffold for cell culture. First, the necessity of surface treatment for SJI-001 was evaluated. In some cases, cell attachment, morphology, and cell proliferation speed on the material surface are improved by plasma treatment [24]. On the other hand, there is a possibility that the material is etched by the plasma, thus damaging it. Therefore, the necessity for plasma treatment of SJI-001 was evaluated by measuring the wettability and etching amount. Next, the effect on cells cultured on SJI-001, which had not been plasma-treated, and extracellular matrix (ECM) coating was evaluated. The adhesion rate and doubling time were measured by seeding HeLa cells into the microchannel, the bottom of which was fabricated with SJI-001. In addition, the effect of SJI-001 was compared to other materials by analyzing surface free energy, calculated from the contact angle. Finally, as a cell culture demonstration, we fabricated a cell culture plate with a 3D lattice structure as a scaffold and evaluated the usefulness of SJI-001 in cell analysis.

## 2. Material and Methods

### 2.1. SJI-001

SJI-001 is a negative type of polymer capable of photolithography [22]. In negative photoresists, the exposed area becomes a solid structure after the development process by a photopolymerization reaction. Because SJI-001 is capable of depositing at a thickness of over 100 µm, making it suitable for fabricating 3D complex microstructures, apart from 2D micropatterns. Compared to SU-8 (KAYAKU Advanced Materials, Inc., Westborough, MA, USA) used as the composition material for micro-electromechanical systems (MEMS), SJI-001 mainly has cyclohexane and phosphorus in place of the benzene ring and antimony. Since SJI-001 does not have a benzene ring that causes auto-fluorescence, the auto-fluorescence of SJI-001 is lower than that of SU-8. The S/N ratio between the background fluorescence of cells and SJI-001 was higher than that of SU-8 because of the former’s low auto-fluorescence. Therefore, SJI-001 is useful for fluorescence imaging.

### 2.2. Surface Modification by Plasma Treatment

Surface modification by plasma treatment, which is generally used as a pretreatment for plastic substrates, was carried out. The contact angle is one of the indexes of wettability and is used for evaluating the effects of surface treatment in various studies [25]. Moreover, because organic polymers, such as photoresist, are etched by plasma, plasma damage is evaluated by measuring the etching amount. In this study, we used SJI-001 and SU-8 samples for comparative experiments.

The SJI-001 sample was fabricated by photolithography. First, the glass substrate was cleaned using a piranha solution. SJI-001 was fabricated by mixing 4 vol. % photoinitiator CPI100P (San-Apro, Ltd., Kyoto, Japan) and 96 vol. % epoxy resin, using a planetary stirring machine (Mazerustar, Kurabo Co.). SJI-001 was spin-coated on the glass substrate at 3000 rpm for 30 s. To volatilize the solvent, the SJI-001 layer was baked at 65 °C for 1 h and 95 °C for 3 h. The SJI-001 layer was exposed to the whole surface by exposure light with a wavelength of 365 nm and energy of 3000 mJ/cm^2^ (PM50C-125A1, Ushio, Inc., Tokyo, Japan). SJI-001 was cross-linked by post-exposure baking (PEB, 65 °C 5 min, 95 °C 5 min). To conduct the same process for fabricating the micropattern, the SJI-001 layer was developed using 1-methoxy-2-propyl acetate(SU-8 Developer, Nippon Kayaku Co., Ltd., Tokyo, Japan) and rinsed with 2-propanol (EL2-propanol, Kanto Chemical Co., Inc., Tokyo, Japan).

For comparison, the SU-8 sample was also fabricated by photolithography. First, the glass substrate was cleaned using piranha solution. SU-8 (SU-8 3005, KAYAKU Advanced Materials, Inc. JP) was spin-coated at 3000 rpm for 30 s. To volatilize the solvent, the SU-8 layer was baked at 65 °C for 5 min and 95 °C for 5 min. The SU-8 layer was exposed to the whole surface by exposure light with a wavelength of 365 nm and energy of 180 mJ/cm^2^. The SU-8 was cross-linked by PEB (65 °C for 5 min, 95 °C for 5 min). To conduct the same process for fabricating the micropattern, the SU-8 layer was developed using 1-Methoxy-2-propyl acetate and rinsed with 2-propanol.

Plasma treatment was conducted by reactive ion etching (RIE, ES401, Nippon Scientific Co., Ltd. JP). The sample was placed in the chamber, with the temperature of the bottom substrate set to 30 °C. Oxygen gas was introduced into the chamber at a flow rate of 2.5 × 10^−6^ m^3^/s. The pressure in the chamber was 66 Pa. Plasma was generated by applying 200 W of electric power between the two electrodes in the chamber. Plasma treatment was conducted from 1 min to 80 min maximally.

The dropping method and measurement of surface roughness were used to calculate the contact angle and etching amount, respectively. In the dropping method, the angle between the dropped pure water and the sample surface was measured using an automatic dynamic contact angle meter (DM-500, Kyowa Interface Science, Inc., Saitama, Japan). The etching amount was measured using a surface roughness tester (130A, Tokyo Seimitsu Co., Ltd., Tokyo, Japan).

### 2.3. Basic Surface Properties for Cell Culture and Analysis

Our research group developed a portable cell culture system for evaluating the effect of material on cells in a closed microfluidic system [26,27]. The developed system included a battery, a peristaltic pump, a microchannel and a reservoir. Cells seeded inside the microchannel were cultured for a long period with a controlled flow rate maintained by the peristaltic pump, which was connected to the microchannel and reservoir. The microchannel culturing cells had a cross-sectional area of width 1 mm and height 100 μm. SJI-001 was used at the bottom of the microchannel. The side and top walls of the microchannel were made of polydimethylsiloxane (PDMS, SILPOT 184, Dow Corning Toray Co., Ltd., Tokyo, Japan). The effect of SJI-001 on adherent cells was evaluated by culturing adherent cells in the microchannel.

HeLa-H2B-GFP cells (HeLa cells), which have a GFP fluorescent protein, were adopted as cultured adherent cells [28]. The medium used in this study was prepared by mixing Dulbecco’s Modified Eagle’s Medium (DMEM, D5796500ML, Sigma, MO, USA) with 10 *v/v*% fetal bovine serum (35–076-CV, Corning, NY, USA) and 0.2 *v/v*% penicillin–streptomycin (P4333-20ML, Sigma, MO, USA). Phosphate-buffered saline (PBS), the concentration of which was 9.55 g/L, was prepared as a cell washing solution by diluting PBS tablet (pH 7.4) (T900, Roman Industries Co., Ltd., Tokyo, Japan). The PBS was sterilized by autoclave the condition of which was 120 °C, 2 h. Trypsin (0.25%) with 1-mM EDTA solution (25200056, Thermo Fisher Science, Waltham, MA, USA) was used as the cell-releasing solution.

To count the number of cells inside the microchannel, the images were taken using a fluorescence microscope (IX71, Olympus Co., Tokyo, Japan) and analyzed using a public domain image processing and analysis program, ImageJ [29]. The magnification and numerical aperture (NA) of the objective lens were 4-times and 0.13 respectively. To avoid the influence of turbulence of flow around the inlet/outlet of the microchannel, a 4 mm^2^ area of the center of the microchannel was used as an observation area. The number of cells in the observation area was counted using ImageJ.

The adhesion rate was calculated by using the difference in the number of inducted cells into the microchannel and the remaining cells after perfusion flow. The proliferation rate was evaluated from the doubling time. The doubling time was calculated by the gradient of the cell proliferation curve, cell number and cell culturing time. Surface free energy, calculated by using contact angles, is one of the criteria for the surface condition of materials. There exists an established correlation between surface free energy and cell adhesion/proliferation rate [26,27]. The surface free energy of each material was calculated using the contact angle between each evaluating material and a liquid whose surface free energy was already known. In this research, pure water and formamide were used as the liquid for this purpose [30]. The surface free energy of each material was calculated by solving simultaneous equations with measured contact angles based on the Kaelble-Uy’s theory [31].

### 2.4. Cell Culture Plate

Scaffolds are necessary for culturing adhesion cells. Scaffolds for cell culture, which are 2D and 3D topographic cues for culturing cells, are composed of various materials and have various shapes, such as solid, flexible, rough surface or porous that are suitable for several cell culturing purposes. In two dimensional cell culture, flat and smooth surface solid materials, such as petri dishes, are used. On the other hand, since the surface roughness affects cells and shapes of cellular tissue, cell culture on the rough surface has also received attention [32]. The surface roughness increases the strength of adhesion and support cell proliferation [33]. There is the opposite effect, depending on the cell, surface roughness reduces the adhesion area of cells and substrate, thus facilitating the formation of spheroids by promoting cell–cell adhesion [34]. As another research example, scaffolds that are thin and flexible have also been proposed [35]. These scaffolds are capable of stretching cells or cellular tissue by changing their shape. In addition, scaffolds with porous membranes are used for creating barrier tissue which controls the permeation of nutrients in the human and animal body. Materials are partially separated by barrier tissue on the porous membrane and permeate to another side partially [36,37]. As shown in these examples, scaffolds are capable of affecting cells and cellular tissue. Consequently, there is much existing research available on scaffolds for cell culture.

In addition, there are some scaffolds with periodic or designed structures fabricated by photolithography. For example, there are freestanding cell culture plates having micropores made of SU-8 since it is capable of fabricating micro-sized structures [38,39,40]. This freestanding cell culture plate is thin and has a rough surface or porous membrane. Because these cell culture plates are thin, the noise becomes small when it is used for fluorescence observation. In addition, the physical cue is given to the shape of the cellular tissue by using the roughness surface. Furthermore, the innate property of the barrier tissue that selects permeable material is mimicked by seeding cells on the porous membrane. In this study, a freestanding cell culture plate was fabricated using SJI-001. We evaluated the thickness, shape and 3D lattice structure of the fabricated cell culture plate, as well as its effect on cultured cells.

**Figure 1:**
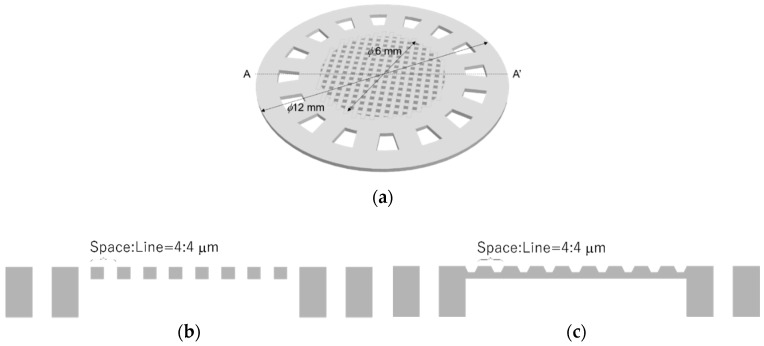
Schematic images of the cell culture plate. The cell culture plate had the 3D lattice structure with a diameter of *ϕ*6 mm. (**a**) 3D lattice structure was supported by the ring structure. The diameter included ring structure was *ϕ*12 mm. The 3D lattice structure fabricated by the mask pattern having 4:4 µm space and line patterns. We designed two types of 3D lattice structures which were (**b**) porous structure and (**c**) the rough surface having no pores. These structures were fabricated by using the same mask pattern and adjusting exposure energy.

The designed cell culture plate had a 3D lattice structure that was a periodic pattern having pores smaller than cells or a rough surface without pores. The 3D lattice structure was supported by a ring structure. Schematic images of the cell culture plate are shown in Figure 1. The diameter of the cell culture plate was *ϕ*12 mm. The center of the cell culture plate had a 3D lattice structure with a diameter of *ϕ*6 mm. The 3D lattice structure consisted of a periodic pattern with a space size of 4 µm and line size of 4 µm. We designed two types of 3D lattice structures: arrayed pores and roughness versus no pores. These two types of 3D lattice structures were fabricated using the same mask pattern. The detailed fabrication process was decided by adjusting the exposure energy.

**Figure 2:**
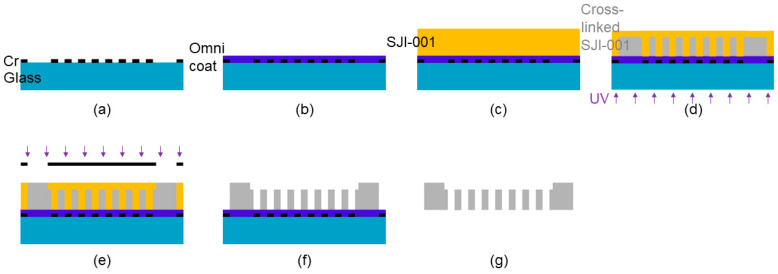
Schematic images of the fabrication process of cell culture plate having the porous 3D lattice structure. The cell culture plate was fabricated by double side exposure to a single SJI-001 layer. The porous or rough surface 3D lattice structure was fabricated via the same fabrication process, with a different exposure energy. (**a**) Spattering Cr layer. (**b**) Coating sacrificial layer. (**c**) Depositing SJI-001 layer. (**d**) Exposing from the back side of the glass substrate. (**e**) Exposing from the top side of the glass substrate. (**f**) Developing uncross-linked SJI-001. (**g**) Peeling off the cross-linked SJI-001.

A cell culture plate made of SJI-001 was fabricated by photolithography with double-side exposure. Edge beads, which is the difference in thickness of the deposited photoresist, make contact gaps and reduce the resolution of the fabricated structure. In addition, the exposure energy for fabricating the micro-scale 3D lattice structure and millimeter-scale ring structure was different. Therefore, we used the double exposure process to fabricate the cell culture plate from a single layer of SJI-001. The schematic images of the fabrication process are shown in Figure 2. First, the 2D lattice pattern was fabricated by the patterned Cr layer, which was deposited by sputtering (E-200S, Canon Anelva Co., Kanagawa, Japan) (Figure 2a). The sacrificial layer (Omnicoat, Kayaku Advanced Materials, Inc., JP) was spin-coated at 1000 rpm for 30 s and baked at 200 °C for 1 min (Figure 2b). To fabricate the SJI-001 layer, the thickness of which was 50 µm, SJI-001 was spin-coated at 1000 rpm for 30 s. To volatilize the solvent, the SJI-001 layer was baked (65 °C for 1 h, 95 °C for 3 h) (Figure 2c). After relaxation for an hour, the SJI-001 layer was exposed to i-line (wavelength 365 nm) using a mask-aligner (PM50C-125A1, Ushio, Inc., Tokyo, Japan) from the posterior surface of the glass substrate (Figure 2d). Next, the SJI-001 layer was exposed to an exposure dose of 3000 mJ/cm^2^ with i-line (wavelength 365 nm) from the top side of the glass substrate to fabricate the ring structure (Figure 2e). The SJI-001 layer was cross-linked by PEB (65 °C for 1 min, 95 °C for 5 min). The SJI-001 layer was developed using 1-Methoxy-2-propyl acetate (SU-8 Developer, Kayaku Advanced Materials, Inc., JP) (Figure 2f). After development, the cell culture plate, which was made of SJI-001, was peeled off by etching the sacrificial layer using tetramethylammonium hydroxide (NMD-3, Tokyo Ohka Kogyo Co., Ltd., Tokyo, Japan) (Figure 2g). After peeling off, the cell culture plate was developed to remove the remaining un-crosslinked SJI-001 resist inside the 3D lattice structure using 1-Methoxy-2-propyl acetate.

To evaluate the cell culture plate with the 3D lattice structure, HeLa cells were cultured on a plate that was not treated with plasma and coated with a protein, such as fibronectin and collagen. We used the same HeLa cells, medium and buffer solution in Section 2.3. First, the cell culture plate was sterilized with 78% ethanol for 30 min and rinsed with PBS. The cell culture plate was sandwiched between two silicone rubber sheets with a thickness of 1.5 mm and an *ϕ*8 mm hole. The *ϕ*8 mm hole would work as a chamber for dropping the cell suspension. The cell culture plate, which was sandwiched between two silicone rubber sheets, was placed into a Petri dish. HeLa cells were cultured in a flask with an area of 25 cm^2^. After HeLa cells became confluent, DMEM was removed. HeLa cells were washed with 10 mL PBS, and then 1 mL of cell releasing solution was added to the flask. After 5 min of incubation, the cell suspension was created by adding 9 mL DMEM. The cell suspension was centrifuged at 900 rpm for 3 min using a centrifugal separator (SRX-201, TOMY SEIKO Co., Ltd., Tokyo, Japan). After removing the supernatant, 10 mL of DMEM was added 200 µL of the cell suspension was dropped into the chamber. After the HeLa cells were cultured in a CO_2_ incubator for 1 d, the cell culture plate was removed from the silicone rubber sheets. HeLa cells not attached to the cell culture plate were removed by washing with PBS. The cell culture plate and HeLa cells, which were attached, were placed in a petri dish with 10 mL DMEM. The fluorescence images of HeLa cells on the cell culture plate were taken 3, 5 days after cell seeding on the cell culture plate using a fluorescence microscope (IX71I, Olympus Co., JP). To remove cells that were not attached to the cell culture plate, the cell culture plate was washed with PBS before each imaging.

## 3. Results

### 3.1. Surface Modification by Plasma Treatment

The surface free energy, measured by using two kinds of liquid, was 36.6 mJ/m^2^ and 27.2 mJ/m^2^ for untreated SJI-001 and SU-8, respectively. The surface free energy of SJI-001 was 1.3-times higher than that of SU-8.

**Figure 3:**
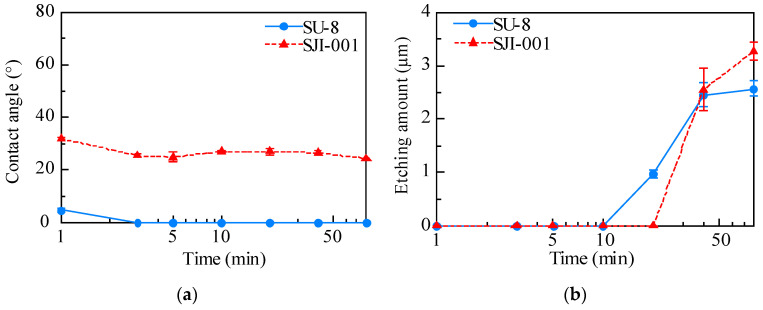
The effect of plasma treatment on SJI-001 and SU-8. The contact angle of both materials was decreased. Although the contact angle of SU-8 was decreased to lower than 5°, the contact angle of SJI-001 stopped declining around 25° (**a**). The etching speed of both materials decreased after 40 min of plasma treatment (**b**).

The surface conditions of SJI-001 and SU-8 were altered by plasma treatment. The contact angle and etching amount of each material are shown in Figure 3. The original contact angle of SU-8 and SJI-001 were 82.3° and 63.2°, respectively. The contact angle was decreased with plasma treatment. In SU-8, the contact angle was lower than 5° after plasma treatment for longer than 3 min. On the other hand, in SJI-001, the contact angle stopped declining at approximately 25°. A similar trend was observed between the etching amounts of SJI-001 and SU-8. Both materials were not etched within 10 min of plasma treatment. Etching speed was increased after 20 min, but decreased after 40 min.

### 3.2. Basic Surface Properties for Cell Culture and Analysis

**Figure 4:**
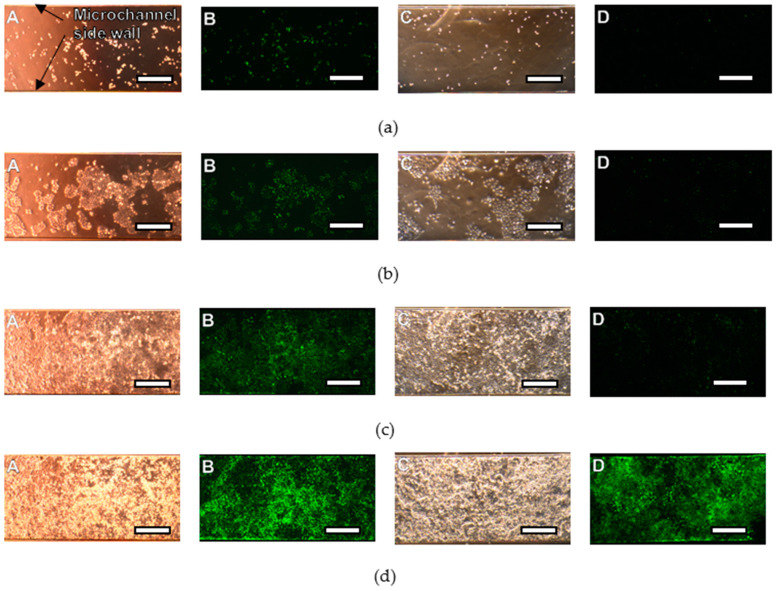
Bright field images and fluorescent images of HeLa cells cultured on SJI-001 and SU-8. HeLa cells were cultured 9 days in PDMS microchannel, the bottom material of which was SJI-001 and SU-8. Bright field images and fluorescence images taken within 2 h after seeding are shown in (**a**). Bright field images and fluorescence images taken 3, 6, 9 days after seeding are shown in (**b**–**d**) respectively. Bright field images and fluorescence images of HeLa cells on SU-8 are shown in A and B, respectively. Bright field images and fluorescence images of HeLa cells on SJI-001 are shown in C and D, respectively. The scale bar is 300 µm.

**Figure 5:**
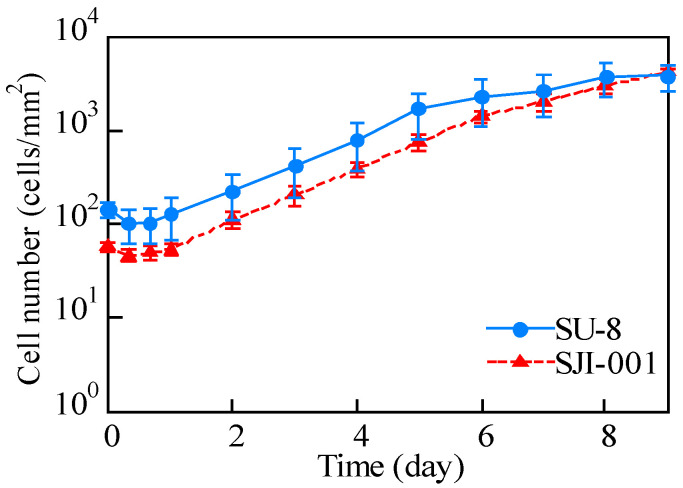
Proliferation curve of HeLa cells on SJI-001 and SU-8. HeLa cells seeded on SU-8 and SJI-001 proliferated and were confluent after 7 and 9 days, respectively.

The adhesion rate and doubling time were measured by culturing HeLa cells on the SJI-001 and SU-8 using a portable cell culture system. HeLa cells on each material were observed within 2 h of seeding. In addition, it was observed 8, 16 and 24 h after seeding. Next, HeLa cells were observed every day, up to 9 days after cell seeding. HeLa cells on each material are shown in Figure 4. The cells shown in “A” and “B” were HeLa cells cultured on SU-8. The cells shown in “C” and “D” were HeLa cells cultured on SJI-001. We measured the background intensity of the fluorescent images taken at 0–2 h after cell seeding by the image. We randomly selected 5 points where there were not cells and measured the intensity of points. The average and standard deviation of background intensity of SU-8 and SJI-001 were 11.0 ± 0.6 and 4.6 ± 0.5, respectively. HeLa cells proliferated on both materials. The number of cells in one unit area was measured using ImageJ. The cell culture time and cell density are shown in Figure 5. The x-axis is the elapsed time, while the y-axis is the number of cells in the unit area. The error bar means the standard deviation of the microchannel culturing tests repeated 5 times. HeLa cells were confluent 7 and 9 days after seeding on SU-8 and SJI-001, respectively. The doubling and adhesion rates are shown in Table 1. The ratio of adhered HeLa cells on SJI-001 was 8.8% larger than that on SU-8. In addition, the doubling time of HeLa cells on SJI-001 was 10.7% shorter than that of SU-8.

### 3.3. Cell Culture Plate

**Figure 6:**
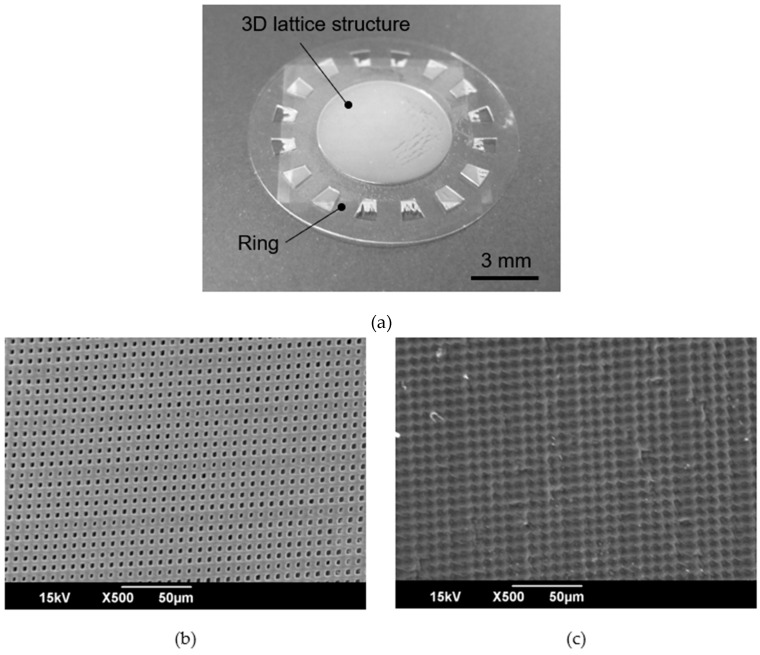
Images of the fabricated cell culture plate. The fabricated cell culture plate had the 3D lattice structure which was the rough surface. The pore, which was not penetrated, on the 3D lattice structure was smaller than the designed size. (**a**) The fabricated cell culture plate having 3D lattice structure. (**b**) SEM image of the 3D lattice structure on the side that was in contact with the Cr pattern on the glass substrate (Top side). (**c**) SEM image of the 3D lattice structure on the side that was not in contact with the Cr pattern on the glass substrate (Back side).

A cell culture plate with a 3D lattice structure made of SJI-001 was demonstrated as one of the scaffolds for cell culture. The cell culture plate fabricated with 1000 mJ/cm^2^ exposure energy for the 3D lattice structure is shown in Figure 6a. There were no breakages on the 3D lattice structure, which was in the micrometer-scale supported by the ring structure, which was in the millimeter-scale. The thickness of the 3D lattice structure and ring structure were 36.18 ± 0.88 µm and 58.53 ± 2.34 µm, respectively. The SEM images of the anterior and posterior sides of the 3D lattice structure are shown in Figure 6b,c, respectively. There were pores with sizes ranging from 2 to 4 µm. On the other hand, all pores were closed even when the exposure energy was changed from 700 to 1000 mJ/cm^2^. In addition, the 3D lattice structure fabricated with exposure energies lower than 800 mJ/cm^2^ had breakages. Therefore, in this experiment, a 3D lattice structure with a rough surface was fabricated, but the porous structure was not fabricated.

**Figure 7:**
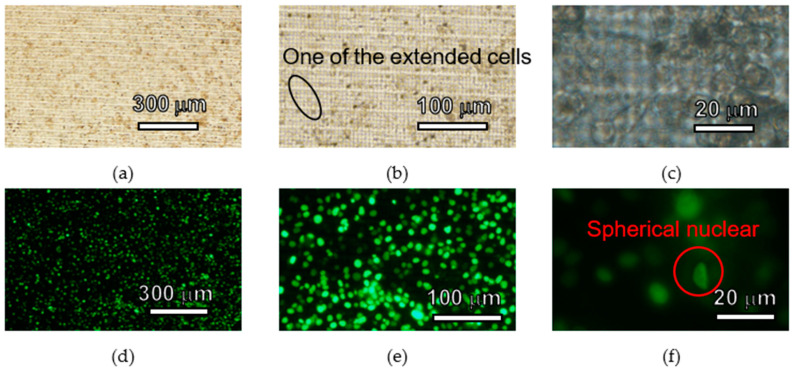
Images of HeLa cells cultured 3 days on the cell culture plate. (**a**) Bright field (×40). (**b**) Bright field (×100). (**c**) Bright field (×400). (**d**) Fluorescent (×40). (**e**) Fluorescent (×100). (**f**) Fluorescent (×400).

**Figure 8:**
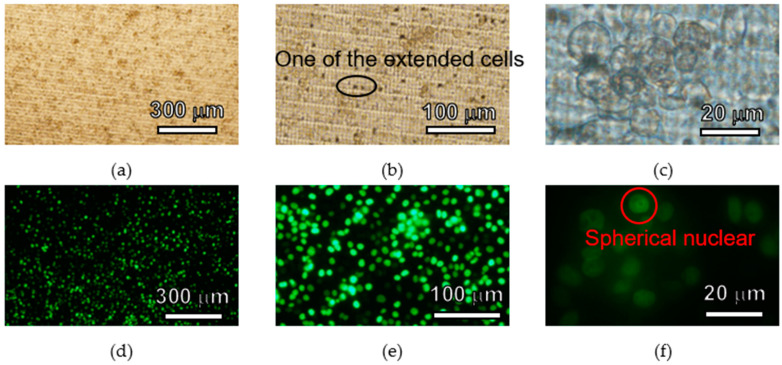
Images of HeLa cells cultured 4 days on the cell culture plate. (**a**) Bright field (×40). (**b**) Bright field (×100). (**c**) Bright field (×400). (**d**) Fluorescent (×40). (**e**) Fluorescent (×100). (**f**) Fluorescent (×400).

**Figure 9:**
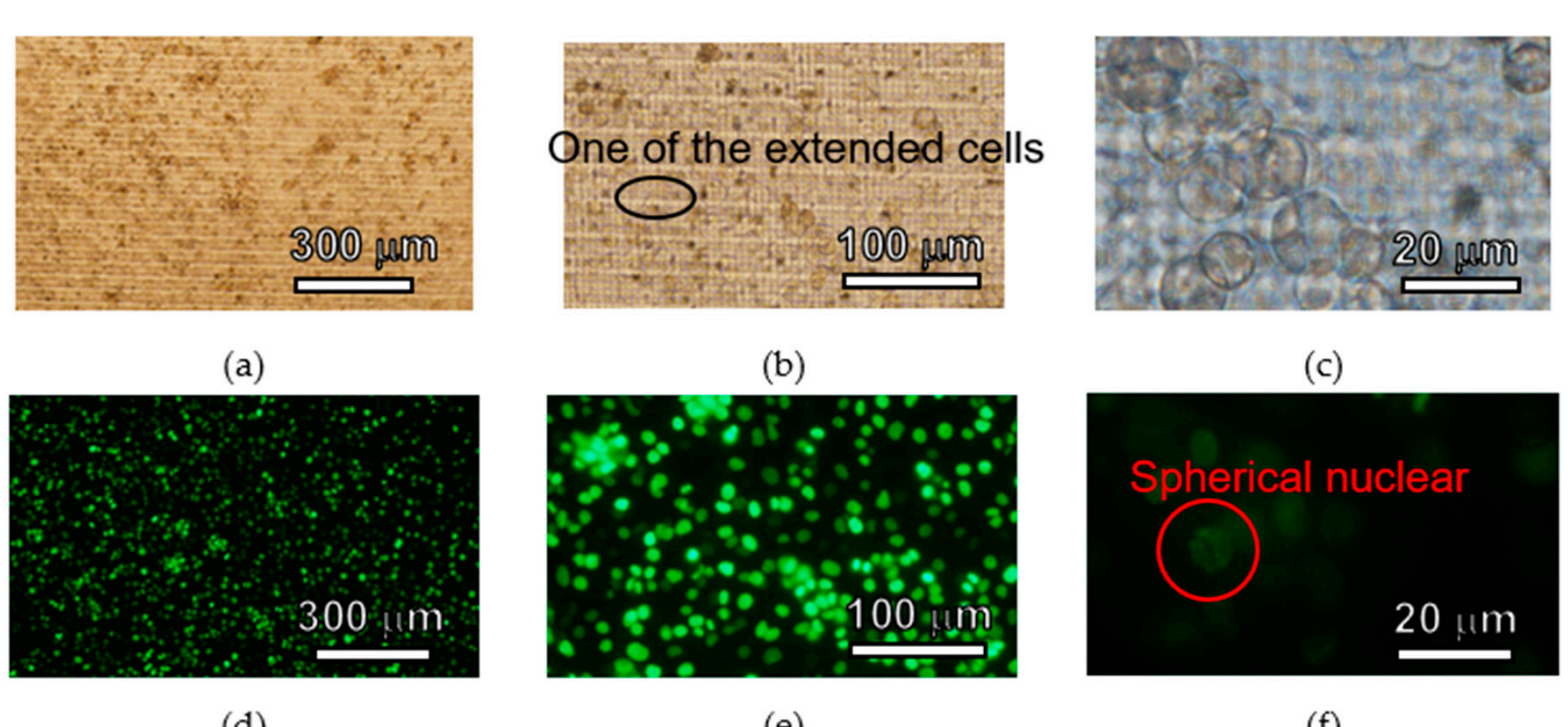
Images of HeLa cells cultured 5 days on the cell culture plate. (**a**) Bright field (×40). (**b**) Bright field (×100). (**c**) Bright field (×400). (**d**) Fluorescent (×40). (**e**) Fluorescent (×100). (**f**) Fluorescent (×400).

HeLa cells were seeded on the 3D lattice structure in the fabricated cell culture plate and cultured for 5 days. Bright field images and fluorescent images of HeLa cells are shown in Figure 7, Figure 8 and Figure 9. In each figure, there are 40-, 100- and 400-times magnification images. HeLa cells were observed in all images and GFP fluorescence was observed. HeLa cells were spread across the entire area of the 3D lattice structure. After 5days, HeLa cells were still attached to the surface of the 3D lattice structure. In all images the magnification of which was 40-times, there are not space over than 100 µm between HeLa cells. In addition, in all images the magnification of which was 100-times, some HeLa cells on the SJI-001 microstructure was extended. In all images with the magnification of 400-times, the nuclear of HeLa cells maintained an almost spherical shape.

## 4. Discussion

### 4.1. Surface Modification by Plasma Treatment

The contact angles of SU-8 and SJI-001 were changed to almost 0° and 25°, respectively. The contact angle of SJI-001 did not decrease below 20°. The contact angle of both materials became constant after 3 min of plasma treatment. On the other hand, the etching amount was not measured within 3 min. Etching amounts were measured after 10 min and 20 min for SU-8 and SJI-001, respectively. This indicated that the surface of SJI-001 was more hydrophobic than SU-8 and was not etched after the wettability became constant.

The contact angle of both materials was decreased because plasma treatment made the hydroxy group on the material surface. However, the minimum contact angle of each material was different. As mentioned above, SJI-001 was different from SU-8 in that the main material was replaced by cyclohexane from the benzene ring, and the component of the photoinitiator was replaced by phosphorus instead of antimony. The reason why the contact angle of SJI-001 did not become 0 ° and the etching amount was smaller than that of SU-8 was the replacement of cyclohexane from the benzene ring. Cyclohexane has an annular shape similar to that of the benzene ring, but it has low reactivity because it is saturated with hydrogen [41]. Therefore, SJI-001 had less plasma damage and higher stability than SU-8.

The plasma treatment for SU-8 was useful for cell culture [24]. The surface of SJI-001 was also treated with plasma. However, the effect of plasma treatment was limited, and it was indicated that the advantage of plasma treatment was also limited. Although the stability of the SJI-001 material was higher than that of SU-8, there is a possibility that the tiny structure may collapse by etching. Therefore, plasma treatment is not necessary for SJI-001 as a pretreatment for cell culture.

### 4.2. Basic Surface Properties for Cell Culture and Analysis

In previous studies, we evaluated the glass substrate (S1111, Matsunami Glass Ind., Ltd., Osaka, Japan), which was cleaned using piranha solution, polystyrene, which was sterilized by ethylene oxide gas and UV irradiation (PS, I-90-20, Ina∙Optica Co., Ltd., Osaka, Japan), SU-8 (SU-8 3005, KAYAKU Advanced Materials, Inc. JP), PDMS (SILPOT 184, Dow Corning Toray Co., Ltd.), Si, which is a semiconductor material and CYTOP(CTM807-AP, Asahi Glass Co., Ltd., Tokyo, Japan), which is an amorphous fluorine plastic [26,27]. There is the correlation between surface free energy and adhesion rate/doubling time even include SJI-001. The surface free energy of SJI-001 was located between the glass and PS. The adhesion rate and doubling time were almost equal to those of glass and PS. Therefore, the SJI-001 was suitable for cell culture experiments as much as other materials that were used for the biologic experiment.

By comparing the two thick photoresists, the adhesion rate and doubling time of SJI-001 were found to be higher and shorter than those of SU-8. Cyclohexane is more stable than the benzene ring, which is composed of SU-8. In addition, the antimony containing SU-8 is a deleterious substance. The effect of antimony containing cross-linked SU-8 on cells or cellular tissue is not well defined. Future research should evaluate the effect of the contained material by measuring not only the doubling time, but also the cell activity, such as metabolism.

### 4.3. Cell Culture Plate

The cell culture plate, fabricated with 1000 mJ/cm^2^, had a 3D lattice structure with a thickness of less than 50 µm and a millimeter-scale ring structure. In addition, there were no breakages. When a thin structure having a microstructure is released from the substrate, it becomes bent by residual stress [42]. The 3D lattice structure and ring structure of the cell culture plate fabricated by SJI-001 were not bent. The temperature of the bake for removing the solvent for SJI-001 and SU-8 was equal. The baking time for SJI-001 was longer than that of SU-8. The cell culture plate made of SJI-001 was not bent even when the baking time was longer. It is indicated that this characteristic is useful for reducing the residential stress when the large size structure in-plane direction and useful for fabricating a wide-scale structure by a batch process.

The fabricated cell culture plate had a 3D lattice structure that had a rough surface and did not have pore which was perforated. A cell culture plate with a rough surface 3D lattice structure was fabricated by a single SJI-001 layer. There was a possibility that the pore was closed because the exposure to light that was irradiated from the backside of the substrate was scattered and lost the verticality. Since the verticality of the fabricated structure was not improved by adjusting the exposure energy, it was found that it was difficult to fabricate the 4 µm pattern in the 50 µm layer. In this research, it was difficult to fabricate a tiny structure with an aspect ratio higher than 10 by using the SJI-001. For fabricating 3D lattice structures with perforable pores smaller than cells for creating barrier tissue, it is required that not only using a thinner layer, but also an additional design because the thinner cell culture plate may not be freestanding.

HeLa cells cultured on the cell culture plate were attached 5 days on the 3D lattice structure and bright field and fluorescence images were obtained. There was no void between cultured cells, which were created by localized cell detachment by washing process of the cell culture plate before observing it. If cells or cell layer which was attached on the microstructure was damaged by the external force due to less cell adhesion strength, there could be some spots which the cells or cell layer were removed. Since there were no over 100 µm square area where there were no cells, it means that cells or cell layer was not physically damaged by external force when the cell culture plate was washed. Hence, the 3D lattice structure of the cell culture plate does not significantly change the adhesion of HeLa cells and has sufficient cell adhesion strength to withstand the normal washing process. If the base material was not suitable for HeLa cells, HeLa cells were not attached and extended [43,44]. Some HeLa cells was extended on the SJI-001 microstructure. Because HeLa cells attached and extended on the SJI-001 microstructure fabricated by photolithography, it was indicated that the SJI-001 microstructure was capable of being used as cell chip for cell culture and cell analysis [44]. The nucleus of HeLa cells had spherical shape. In the terms of necrosis, nucleus of cells collapsed and lost the spherical shape. HeLa cells on the SJI-001 microstructure was kept good condition until 5 days after seeded.

As described above, in the demonstration, it was observed that the HeLa cells were adhered and proliferated on the cell culture plate. In addition, the SJI-001 used in this research is characterized not only high-resolution, but also low-fluorescence. In Figure 7, Figure 8 and Figure 9, the bright field images show that the 3D lattice structure of the SJI-001 and cells are visible, but the fluorescence images show that only the nucleus of the cell is visible. Since the S/N ratio changes depending on the condition of observation, quantitative evaluation is difficult. However, it is indicated that there is a sufficient S/N ratio in GFP fluorescence observation of the HeLa cells. In addition, the wavelength spectrum of the quantitative absorption is shown in our previous research [22]. Therefore, the S/N ratio is also increased in other wavelength and the sharpness of fluorescent imaging is also improved.

In future research, cell cultures using other kinds of cells should be conducted, as HeLa cells are robust compared to other cells. There is the possibility that other kinds of cells are able to be cultured on the SJI-001 because Wang et al. [38] cultured HeLa, HepG2, HUVEC, NIH-3T3 on SU-8. In order to use cell culture plates for long-term cell culture, the metabolism of various types of the cells cultured on the cell culture plate should be evaluated by measuring albumin or urea.

## 5. Conclusions

In this study—to evaluate SJI-001—we measured the changing wettability of SJI-001, cultured adhesion cells on SJI-001 and demonstrated that the cell culture plate with a 3D lattice structure functions as a cell culture scaffold. The surface of SJI-001 was unchanged by plasma treatment compared to SU-8, indicating that SJI-001 was a more stable material than SU-8. Adhesion rates and doubling times of adherent cells on SJI-001 were higher and shorter than those on SU-8, respectively. Since the cell culture plate with the 3D lattice structure was freestanding, it was indicated that the cell culture plate was useful for fluorescence imaging, which had a high S/N ratio and high magnification. HeLa cells seeded on the cell culture plate were cultured for five days and some of them were affected by microstructure on the cell culture plate. Since cells on the microstructure made of SJI-001 were affected and observed, the SJI-001 was useful for fabricating cell chips for cell culturing and cell analysis.

## Figures and Tables

**Table 1:** Adhesion rate and doubling time of HeLa cells on S SU-8 and SJI-001.

	SU-8	SJI-001
Adhesion rate (%)	68	74
Doubling time (h)	27.9	25.2
